# Left-sided pancreatic incidentalomas treated with laparoscopic approach: a report of 20 cases

**DOI:** 10.1186/s12957-016-0949-7

**Published:** 2016-08-04

**Authors:** Marco Chiarelli, Martino Gerosa, Fulvio Tagliabue, Luca Fumagalli, Angelo Guttadauro, Francesco Gabrielli, Alessandro Marando, Matilde De Simone, Ugo Cioffi

**Affiliations:** 1 Department of Surgery, Ospedale Alessandro Manzoni, Lecco, Italy; 2 Department of Surgery, University of Milan-Bicocca, Istituti Clinici Zucchi, Via Zucchi, Monza, MB Italy; 3 Department of Pathology, Ospedale Alessandro Manzoni, Lecco, Italy; 4 Department of Surgery, Istituto Clinico Humanitas Mater Domini, Castellanza, VA Italy; 5 Department of Surgery, University of Milan, Milan, Italy

**Keywords:** Pancreatic incidentaloma, Distal pancreatectomy, Pancreas, Laparoscopic surgery

## Abstract

**Background:**

The diffusion of cross-sectional imaging has recently permitted the detection of an increasing number of incidentalomas localized in the distal pancreas.

Currently, there are no studies in the literature exploring the laparoscopic approach as treatment for left-sided pancreatic incidentalomas.

**Methods and results:**

We report a series of 20 incidentalomas localized in the body and tail of the pancreas treated with laparoscopic surgery over the period 2010–2014. The incidental masses of our series included a great variety of histotypes and a relevant proportion of malignant lesions. In two cases, the laparoscopic procedures were converted to open surgery. No postoperative death was observed. The postoperative pancreatic fistula rate was 20 %, and the new-onset diabetes rate was 25 %.

**Conclusions:**

Left-sided pancreatic incidentalomas in patients with minor comorbidities can be safely treated with laparoscopic approach. Only clinical trials will confirm whether laparoscopic surgery is an effective treatment for malignant lesions.

## Background

Pancreatic incidentalomas (PIs) are asymptomatic masses accidentally diagnosed by radiological, endoscopic, or laboratory exams performed for symptoms not suggesting pancreatic diseases [1]. Masses confined to the body and tail of the gland are frequently asymptomatic, but recently, an increasing number of these lesions has been detected, due to the large use of cross-sectional imaging [2]. To date, only few series of PIs have been reported in the literature, and consequently, some aspects of their management are still debated [3–7].

Laparoscopic distal pancreatectomy (LDP) is actually considered an effective and safe treatment for benign and premalignant left-sided pancreatic tumors [8–11]. Minimally invasive surgery could be a good choice for the treatment of incidental masses of the distal pancreas, but currently, there are no studies in the literature confirming this hypothesis.

We report a series of 20 incidentalomas localized in the body and tail of the pancreas treated with laparoscopic approach.

## Methods

After obtaining local ethics committee approval, we retrospectively reviewed the medical records of all the patients who underwent distal pancreatectomy at the General Surgery Division of Alessandro Manzoni Hospital, from January 2010 to December 2015. Our institution is categorized as a medium-volume hospital for pancreatic surgery [12]. Only patients with asymptomatic incidentally identified lesions treated with laparoscopic approach were included into the study. The data collected were as follows: preoperative data—age, gender, ethnicity, body mass index (BMI), American Society of Anesthesiologists (ASA) physical status classification, indication and type of imaging exams, and tumor size and location; intraoperative data—type of resection and operative time; pathological diagnosis and staging (according to the 7th edition of American Joint Committee on Cancer TNM staging system); and postoperative outcomes—perioperative mortality, length of hospital stay, readmission, postoperative pancreatic fistulas (POPF) [13], post-pancreatectomy diabetes (PPD) [14], and generic complications.

LDP was performed according to the standard technique described in the literature [15]. The pancreas was transected with a linear cutting stapler (Endopath^®^ ETS Linear Cutter—Ethicon Endo-Surgery Inc., Cincinnati OH, USA); no extra suture was performed routinely. One suction drain was left close to the transected pancreas.

In patients with malignant neoplasm, the radiological follow-up consisted in CT scan every 6 months. Informed consent for publishing personal data was obtained from each patient included in the study.

## Results

We retrospectively collected 34 cases of tumors of the distal pancreas that underwent surgery during the period 2010–2015: 22 patients (64.7 %) were asymptomatic and 20 (58.8 %) were treated with laparoscopic approach. The mean age was 63.4 years (range 26–78). Fourteen patients were female and 6 male. All patients were Caucasian. The median BMI was 24.75—interquartile (IQ) range 23.7–26.2. Four patients were classified ASA 1 (20 %), 12 patients ASA 2 (60 %), and 4 patients ASA 3 (20 %). The main radiological, pathological, and surgical characteristics of the series are summarized in Tables 1 and 2.

**Table 1:** Demographic, radiological, and pathological data of 20 cases of left-sided pancreatic incidentalomas treated with laparoscopic approach

No.	Age	Sex	BMI	ASA score	Imaging	Location	Size (cm)	Histology
1	69	F	21.4	2	CT-MRI	Body	4.5	SCN
2	39	F	22.9	1	US-CT	Body-tail	9.0	SPPN
3	62	M	25.7	1	CT	Tail	1.5	NET
4	65	M	23.7	2	CT-MRI	Body	3.5	IPMN
5	55	F	23.9	2	CT-MRI	Body-tail	4.5	MCN
6	75	F	26.1	3	CT	Tail	3.8	ACC
7	72	M	32.6	2	CT	Body	5.4	DAC + UC
8	72	F	23.7	2	US-CT	Body	4.5	DAC
9	71	F	25.8	2	CT-EUS	Body	3.7	ACC
10	61	F	24.8	2	CT-MRI	Tail	3.5	SCN
11	26	F	24.2	1	US-CT-MRI	Tail	9.5	SPPN
12	72	F	31.4	3	CT-MRI	Tail	2.4	IPMN + DAC
13	76	F	24.7	2	CT-EUS	Body	1.6	DAC
14	49	F	22.3	1	CT-EUS	Tail	2.5	NET
15	78	F	27.3	3	US-CT-MRI	Body-tail	6.2	MCN
16	52	M	27.8	2	CT	Tail	5.3	CNET
17	75	M	24.7	3	CT-EUS	Body	2.5	DAC
18	67	F	26.3	2	US-CT	Tail	1.8	NET
19	65	M	24.8	2	CT-EUS	Tail	3.0	DAC
20	67	F	21.5	2	CT-MRI	Body	4.5	CNET

*BMI* body mass index, *ASA* American Society of Anesthesiologists, *US* ultrasonography, CT computed tomography, *MRI* magnetic resonance imaging, *EUS* endoscopic ultrasound, *SCN* serous cystic neoplasm, *MCN* mucinous cystic neoplasm, *SPPN* solid pseudopapillary neoplasm, *IPMN* intraductal papillary mucinous neoplasm, *NET* neuroendocrine tumor, *CNET* cystic neuroendocrine tumor, *ACC* acinar cell carcinoma, *DAC* ductal adenocarcinoma, *UC* undifferentiated carcinoma LDP

**Table 2:** Surgical and follow-up data of 20 cases of left-sided pancreatic incidentalomas treated with laparoscopic approach

No.	Surgery	Operative time (min)	Postop stay	Complications	Follow-up
1	LDP	204	7	–	AD
2	LDP	215	10	P, PE	AD
3	LSPDP	228	5	–	AD
4	LDP	220	12	POPF	AD
5	LDP	217	10	P, PE	AD
6	LDP	210	5	–	D
7	LDP-CO	235	8	PPD	D
8	LDP	191	11	POPF, PE	D
9	LDP	188	9	PPD	D
10	LDPDP	198	5	–	AD
11	LDP	162	6	–	AD
12	LDP	238	8	PPD	PD
13	LDP	172	14	POPF	D
14	LDPDP	197	5	–	AD
15	LDP-CO	212	7	PPD	AD
16	LDPDP	230	6	–	AD
17	LDP	203	22	POPF, PE	D
18	LDP	185	6	–	AD
19	LDP	195	7	–	AD
20	LDP	200	9	PPD	AD

*LDP* laparoscopic distal pancreatectomy, *LSPDP* laparoscopic spleen-preserving distal pancreatectomy, *CO*, converted to open, *PE* pleural effusion, *POPF* postoperative pancreatic fistula, *P* pneumonia, *PPD* post-pancreatectomy diabetes, *AD* absence of disease, *PD* progression of disease, *D* died

In 12 patients, diagnosis was made during diagnostic work-up in the emergency department (ED). In three patients, the suspected diagnosis was pulmonary embolism; in two, colonic diverticulitis; and in one, pericarditis. In three patients presenting with hematuria and three with renal stones, PI was an incidental finding.

In six cases, imaging was performed for follow-up (two for pelvic cyst, two for uterine carcinoma, and one for prostate carcinoma) or for staging (one for oral neoplasm). In two patients, PI was detected due to an increase of serum amylases.

Radiological diagnosis was made by abdominal ultrasonography (US) in 5 patients and computed tomography (CT) scan in 15 patients. In nine cases, CT was performed in ED.

In two cases, the laparoscopic procedures were converted to open surgery for the large size of PI. The median operative time was 203.5 min (IQ range 193–218.5). The median postoperative hospital stay was 7.5 days (IQ range 6–10). Four patients (20 %) developed POPF: 2 were grade A and 2 grade B fistulas. No emergent reintervention was required and conservative management was adopted in all cases. Two patients with grade B fistulas were treated with enteral nutrition and antibiotic therapy; the drainage was maintained in place until leakage resolution. In one case, the patient was discharged with drain in situ and reevaluated in an outpatient setting. In our series, the median persistency of POPF was 16 days. In 4 cases (20 %), postoperative pleural effusion was observed: in 2 patients, it was associated with grade B pancreatic fistulas, and in 2 cases, it was secondary to pneumonia; one patient required thoracentesis. Five patients (25 %) developed PPD during the postoperative course. In our series, no case of postoperative death or readmission was observed at 90-day follow-up.

In 8 patients (40 %), the PI was located in the body of the gland; in 9 patients (45 %), in the tail; and in 3 patients (15 %), between the body and tail.

In all the patients, the resection margins were negative for tumor involvement. Histology revealed ductal adenocarcinoma (DAC) in six patients—associated with undifferentiated carcinoma and intraductal papillary mucinous neoplasm (IPMN) in two patients; neuroendocrine tumor in five patients (two presented the cystic variant); and two cases of acinar cell carcinoma (ACC). Serous cystic neoplasm was detected in two patients, mucinous cystic neoplasm in two patients, solid pseudopapillary neoplasm in two patients, and one patient showed an isolated IPMN.

The mean follow-up of the cohort was 31 months. All the patients with cystic neoplasms or neuroendocrine tumors were alive without disease recurrence in December 2015. In 8 patients with DAC and ACC, the median number of lymph nodes removed was 15 (IQ range 13.5–17). In this specific sub-group, pathological staging was stage I B in one patient, stage II A in four patients, and stage II B in three patients. The median follow-up time of this sub-group was 17 months: six of eight patients died of tumor recurrence, while two patients are alive in December 2015 (one with disease recurrence).

## Discussion

**Fig. 1:**
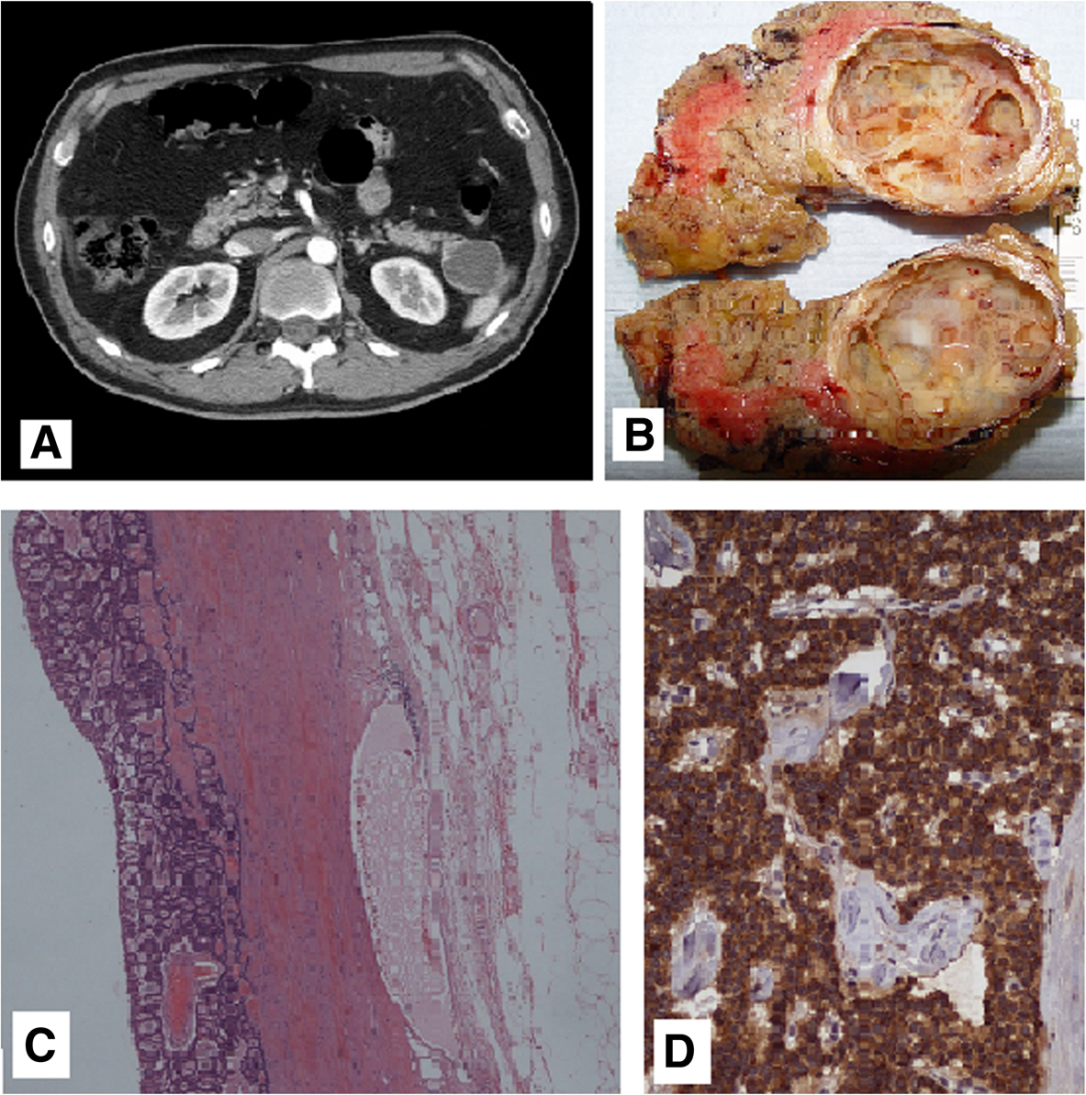
Case 16. **a** CT scan demonstrating a 3 × 4 cm pancreatic tail cystic lesion. **b** Surgical specimen of spleen-preserving distal pancreatectomy with a cystic lesion of the tail. A well-demarcated, solitary, and cystic mass of the pancreatic tail is a rare macroscopic presentation of a neuroendocrine tumor. **c** The characteristic trabecular and gyriform pattern of a pancreatic neuroendocrine tumor with relatively uniform cells (hematoxylin-eosin; magnification ×40). **d** The immunohistochemical staining shows strong and diffuse expression of chromogranin A (magnification ×200)

**Fig. 2:**
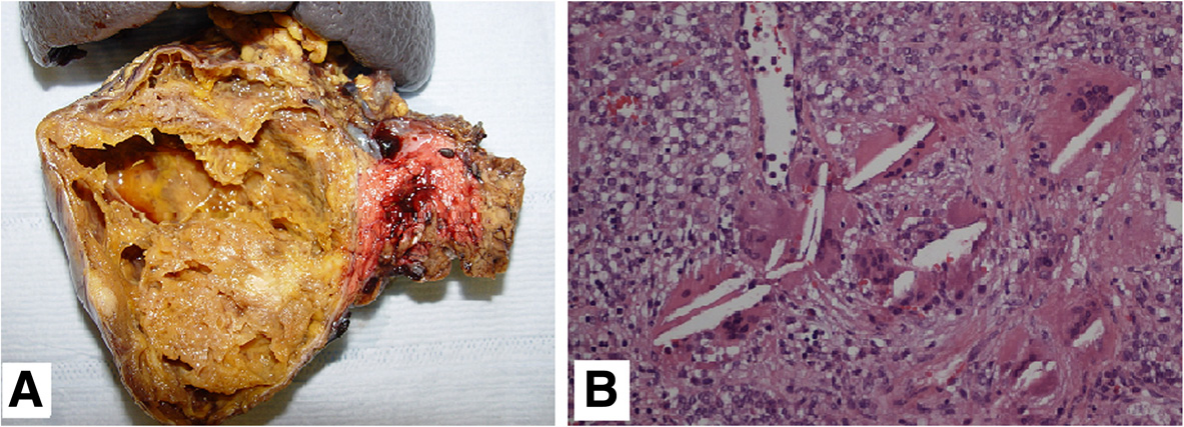
Case 2. **a** Surgical specimen of distal pancreatectomy containing a 9.5-cm-large encapsulated pancreatic tail mass with areas of cystic degeneration. The histological diagnosis was solid pseudopapillary neoplasm. **b** The microscopic pattern of the neoplasm is solid and pseudopapillary with poorly cohesive monomorphic cells, admixed with thin-walled blood vessels. At the center of the image, there are characteristic cholesterol crystals, surrounded by foreign-body giant cells (hematoxylin-eosin; magnification ×200)

**Fig. 3:**
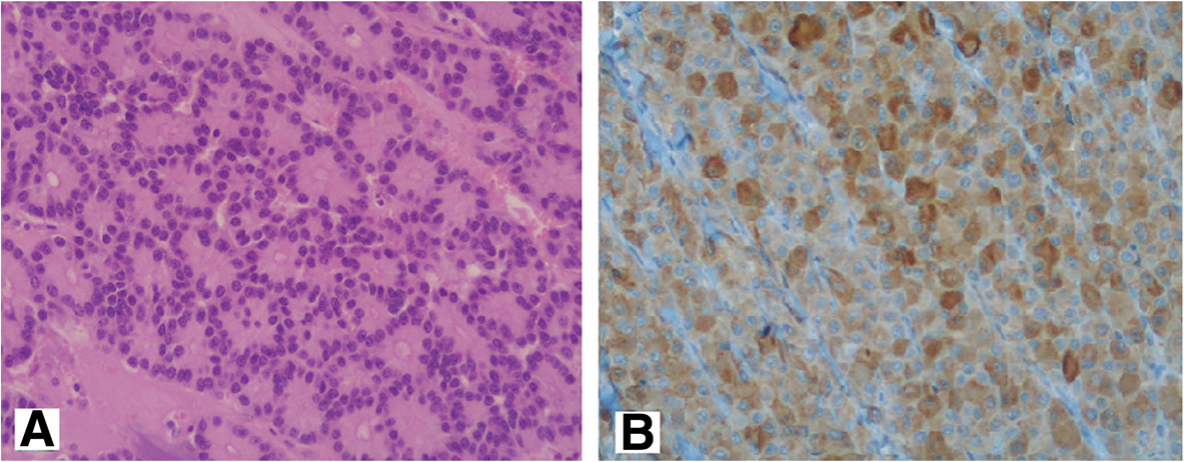
Case 6. **a** Histological picture of an acinar cell carcinoma: the neoplasm composed by cells arranged in small acinar units (hematoxylin-eosin; magnification ×200). **b** The immunohistochemical staining proves the expression of pancreatic exocrine enzymes (trypsin; magnification ×200)

The preoperative work-up of a pancreatic lesion should determine its nature in order to plan the most accurate treatment. Nevertheless, in some cases PIs are not easy to characterize preoperatively [2]. Incidental masses of the pancreas include a great variety of lesions and unusual histotypes are frequently counted in the series present in the literature [4, 5, 7]. Differently from DAC, uncommon histological types with a lower biological aggressiveness, such as mucinous cystic neoplasms or neuroendocrine tumors, are preferentially located in the distal pancreas [16–18]: in our series, we found two ACC, two cystic neuroendocrine tumors, and two solid pseudopapillary neoplasms (Figs. 1, 2, and 3). Furthermore, in about 7 % of patients with a pancreatic mass is not possible to establish a definitive diagnosis before surgery despite a complete preoperative imaging [19].

The diagnostic work-up of our series was characterized by the exiguity of preoperative exams. Solid masses localized in the distal pancreas are very frequently suitable for surgical resection, and consequently, CT findings are sufficient to plan the more appropriate management of a left-sided incidentaloma in the majority of the cases [7, 20].

All patients underwent a contrast-enhanced multidetector CT scan with a biphasic protocol (arterial and venous phases). Currently, intravenous contrast-enhanced CT scan is considered the pivotal radiological technique for the detection, definition, and staging of pancreatic masses [21–23]. In the last decade, the use of CT imaging increased by threefold in emergency-treated patients [24]: in our series, eight cases of PI were discovered by a CT scan performed in patients evaluated in the ED.

A relevant proportion of PIs were detected by transabdominal US. US is considered the first-level imaging in hepato-biliary disease, but the deep location of the pancreas and the operator dependency make the US an exam with low accuracy for the correct assessment of a pancreatic tumor [21].

Magnetic resonance imaging (MRI) and endoscopic ultrasound (EUS) were performed in selected cases. A complete pancreatic MRI study including pre- and post-gadolinium T1-weighted sequences, T2-weighted sequences, and magnetic resonance cholangiopancreatography was performed for the definition of the local extension of poorly defined solid masses and the differentiation of cystic neoplasms [22, 23, 25].

EUS with fine-needle aspiration (FNA) provides high-resolution images and relevant information about cytology and tumor markers of solid and cystic lesions, but the high operator dependency is the limit of the exam [21, 22]. EUS presents a high accuracy for small solid tumors, and EUS-FNA may be useful in the differential diagnosis of pancreatic cystic masses [25–27]. Over recent years, we have employed EUS more frequently in the diagnostic work-up of PIs.

Surgical resection is considered the standard treatment for asymptomatic pancreatic solid neoplasms [1, 6]. Conversely, the management of cystic PIs is generally conservative due to the benign nature of the majority of these lesions [2]. Serous cystic neoplasm (SNC) is considered a benign lesion, and surgery should be considered only in case of large tumors (size >4 cm) or when preoperative exams are not conclusive [28]. Mucinous cystic neoplasms (MCN) and IPMN are frequently incidental [25]. Surgical resection is mandatory for MCN and main duct-type IPMN [29]. Branch duct-type IPMN should be considered for surgery only in case of a lesion greater than 3 cm associated with main duct dilatation >10 mm or an enhanced solid component [29].

A relevant proportion of PIs are malignant or premalignant lesions [6, 7]. Malignancies detected incidentally are diagnosed in earlier stages, and long-term survivals seem to be more favorable than symptomatic lesions [7]. In our series, in six cases the histologic diagnosis of left-sided PI was DAC, and in two patients, it was ACC. Despite a complete preoperative diagnostic work-up, the final pathological staging demonstrated a relevant proportion of tumors with infiltration of surrounding tissues or microscopic lymph node involvement: four patients were classified as stage II A and three patients as stage II B. Consequently, when a pancreatic mass is detected, surgical treatment should be always considered [20].

The distal pancreatectomy consists in the resection of body and tail of the pancreas including the spleen and regional lymph nodes: it is considered the standard treatment of left-sided malignancies. Minimally invasive surgery was introduced for DAC staging, and subsequently, it was employed for pancreatic resections [8, 15]. LDP is currently considered an effective treatment for benign and low-grade malignant lesions of the distal pancreas, but it is still debating if this technique is an appropriate treatment for DAC [8–11]. In the series presented in the literature, LDP was performed for non-malignant lesions in the majority of cases, but there are not clinical trials comparing long-term survivals of patients with DAC treated with laparoscopic or open approach [11]. In the meta-analysis of the literature, the tumor free margin rate and number of lymph nodes dissected are comparable in both techniques, but no definitive conclusions can be drawn about outcomes of laparoscopic resection for malignancy [9, 10]. However, LDP presents lower blood loss, shorter time to oral intake, and reduced length of hospital stay as compared to open surgery, while the rate of postoperative pancreatic fistulas is similar for the two surgical techniques [8–10].

We consider the presence of lymph node involvement and infiltration of surrounding tissues at preoperative work-up as contraindications to laparoscopic approach. In our series, conversion to open surgery was determined by the intraoperative detection of considerable-size masses causing the failure to progress. Laparoscopic spleen-preserving distal pancreatectomy was performed in two patients for small benign lesions localized in the tail. In patients with diagnosis of DAC, the number of lymph nodes removed was adequate for a correct oncologic resection.

The absence of postoperative deaths could be related to the epidemiologic features of our cohort: the series was composed by relatively young patients without considerable comorbidities.

In our series, the postoperative fistula rate was slightly high, but it was still in the range reported in the literature [10, 30]. No grade C fistula was observed, and in two cases, the pancreatic leak was only biochemical without any clinical relevance. Our high POPF rate may be attributed to lack of oversewing of the pancreatic stump, but any conclusions about the correlation between the type of surgical closure of the remnant gland and POPF cannot be drawn due to the limited number of the series.

Pleural effusion, a rare complication [8], was observed in four patients. In two cases, pleural effusion was associated with grade B pancreatic fistulas; in two cases was secondary to pneumonia, and it was self-limiting. Pleural and pulmonary complications are considered infrequent complications in pancreatic surgery, but a large observational study shows how LDP is associated with a pleuro-pulmonary morbidity rate of 26 % [11].

The incidence of PPD was consistent with the data of the literature [31].

## Conclusions

In conclusion, circumscribed incidentalomas of the distal pancreas in patients with minor comorbidities could be safely treated with laparoscopic approach. Left-sided PIs are frequently uncommon pancreatic neoplasms, but in a significant share of patients, DAC in early stages are found. In case of large-sized tumors or lymph node involvement, open surgery should be considered. Only clinical trials will confirm whether laparoscopic surgery is an effective treatment for malignant lesions [11].

## Abbreviations

ACC, acinar cell carcinoma; ASA, American society of anesthesiologists; BMI, body mass index; CT, computed tomography; DAC, ductal adenocarcinoma; ED, emergency department; EUS, endoscopic ultrasound; FNA, fine-needle aspiration; IPMN, intraductal papillary mucinous neoplasm; LDP, laparoscopic distal pancreatectomy; MCN, mucinous cystic neoplasm; MRI, magnetic resonance imaging; PIs, pancreatic incidentalomas; POPF, postoperative pancreatic fistulas; PPD, post-pancreatectomy diabetes; SCN, serous cystic neoplasm; US, ultrasonography
